# Risk factors and prognosis of spinal cord injury without radiological abnormality in children in China

**DOI:** 10.1186/s12891-022-05393-8

**Published:** 2022-05-06

**Authors:** Jianmin Liang, Linyun Wang, Xiaosheng Hao, Guangliang Wang, Xuemei Wu

**Affiliations:** 1 Department of Pediatric Neurology, First Hospital of Jilin University, 1 Xinmin Street, Changchun, 130000 Jilin Province China; 2 Jilin Provincial Key Laboratory of Pediatric Neurology, Changchun, China; 3 Department of Cardiology, Jiren Hospital of Far Eastern Horizon, Anda, P. R. China

**Keywords:** SCIWORA, ASIA, Dance practice, Risk factors, Prognosis

## Abstract

**Background:**

Compared to adults, spinal cord injury without radiographic abnormality (SCIWORA) is more common in children due to the congenital spinal soft tissue elasticity and immature vertebral bodies. In this study, we aimed to investigate the risk factors and prognosis associated with SCIWORA in China.

**Method:**

We retrospectively examined patient records at the First Hospital of Jilin University from January 2007 to December 2020. Patients diagnosed with SCIWORA were included in the study group (*n*=16). The age, gender, history of trauma, symptoms, injury level of the spinal cord, the American Spinal Injury Association (ASIA) impairment score according to the International Standards for Neurological Classification of Spinal Cord Injury (ISNCSCI), as well as laboratory and imaging findings were analyzed.

**Result:**

The study group included 16 patients with SCIWORA with a mean age of 6.69±2.51 y. The ISNCSCI impairment scale was significantly different between the pre-school age patients (≤7 years old) and school age patients (>7 years old) before (*P=0.044*) and after therapy (*P=0.002*). Similarly, magnetic resonance imaging demonstrated a significant difference in the spinal injury level between pre-school age and school age patients (*P*=0.041). Further, the study group was subdivided into three subgroups according to the cause of trauma: Dance, Taekwondo, or Falls. Magnetic resonance imaging revealed significant differences among the three subgroups (*P*=0.041).

**Conclusion:**

Compared to school-age patients, pre-school-age patients were more vulnerable to SCIWORA with more severe ISNCSCI scores. Dance and Taekwondo are among the risk factors associated with SCIWORA in Chinese children.

## Background

Spinal cord injury without radiographic abnormality (SCIWORA), first proposed by Pang and Wilberger in 1982, is a spinal cord injury caused by external forces without signs of spinal fracture or dislocation on X-rays or computed tomography (CT) examination [1, 2]. SCIWORA accounts for 6% - 19% of traumatic spinal cord injury in children. Even after timely treatment, the disability rate following spinal cord injuries is relatively high, which leads to a decreased quality of life [3–5] Due to anatomical features, SCIWORA is more common in children than adults. The greater elasticity of children's ligaments and joint capsules enables them to withstand considerable stretching without tearing [6]. Further, the high water content of the intervertebral discs enables their longitudinal extension without fracture [7, 8]. The vertebral body in children is not fully ossified and is wedge-shaped, resulting in a significantly larger spinal range of motion than that in adults [3, 7]. However, the cartilage endplate is weak; therefore, it can be damaged by minor shear forces. The uncinate process that restricts lateral and rotational movements of the vertebra is not yet developed in children under 10 years old. Owing to the above-mentioned pathophysiological reasons, children can easily suffer from spinal cord injuries [4].

The clinical manifestations and imaging results following SCIWORA are not always correlated [9]. This can potentially delay the diagnosis of SCIWORA in children and thus result in the delay of early treatment. Due to the low incidence rate of SCIWORA, published research and clinical evidence are scarce, which negatively impacts SCIWORA diagnosis and therapy. In this study, we retrospectively analyzed the clinical information for children diagnosed with SCIWORA to explore the clinical and prognostic characteristics associated with the injury. The results obtained from this study will improve our understanding of the diagnosis and treatment of SCIWORA in children.

## Methods

### Participants

In this retrospective study, we screened patient records from January 2007 to December 2020 at The First Hospital of Jilin University. For each record, we analyzed the patient’s history, injury symptoms, and laboratory and spinal cord Magnetic Resonance Imaging (MRI) results. Patients ranging from 0-15 years old who were diagnosed with a spinal cord injury at the cervical, thoracic, or lumbar vertebral levels after mild trauma without radiographic evidence of spinal fracture or dislocation were determined to have SCIWORA and enrolled in the study group. Next, the study group was divided into two subgroups according to the age: pre-school age (≤7 years old) and school age (>7 years old). Additionally, the study group was divided into three subgroups according to the cause of trauma: Dance, Taekwondo, or Falls. The experimental protocol was approved by the ethics committee of the First Hospital of Jilin University (2020-686), and all methods were carried out in accordance with relevant guidelines and regulations. Informed consent was obtained from the parents.

### Procedures

Information regarding the age, gender, history of trauma, signs, and symptoms for all patients were collected and analyzed. We assessed and classified the damage to the spinal cord according to the American Spinal Injury Association International Standards for Neurological Classification of Spinal Cord Injury (ISNCSCI )[10, 11]. The family history, developmental stage, and laboratory examination and spinal cord MRI findings were recorded. Further, we also performed routine blood, C-reactive protein, liver function, blood lipid, myocardial enzyme, blood ion, blood glucose, cerebrospinal fluid, spinal cord CT, and MRI assessments. These assessments were performed within 1-2 days from admission.

### Statistical analysis

Data are expressed as means ± standard deviation (SD). Comparisons between different parameters were performed using the analysis of variance (ANOVA and Chi square test) with the Yate's correction. Statistical analyses were carried out using SPSS (version 18.0) statistical software (Chicago, IL, USA). A P value < 0.05 was considered statistically significant.

## Results

### Demographic characteristics and clinical features

Sixteen patients with spinal cord injury without fracture or dislocation due to trauma from January 2007 to December 2020 were included in the study group. The study group included 15 girls and one boy with a mean age of 6.69±2.51 years (3 to 13 years old). The pre-school age (≤7 years old) group included 11 cases, while the school age (>7 years old) group included five cases. The demographic characteristics and clinical features of the study group are listed in Table 1.

**Table 1:** Clinical features of the study group

Cases	Gender	Age (Y)	Cases of Trauma	Latent Period (min)	Level	MRI Location	ISNCSCI	ISNCSCI After Therapy
1	F	4	Dance	240	T12	normal	A	A
2	F	7	Dance	10	T10	T7-L2	A	C
3	F	9	Taekwondo	5	T10	T2-T10	A	A
4	F	3	Falls	30	T12	T5-T12	B	D
5	F	7	Dance	30	T10	T4-T12	D	E
6	F	7	Falls	20	T12	T9-T12	A	A
7	F	9	Dance	20	T4	C7-T5	B	C
8	F	5	Dance	120	T8	T8-T12	A	A
9	F	5	Dance	10	T8	T8-T12	C	D
10	F	8	Dance	5	T12	T11-L2	D	E
11	F	13	Taekwondo	120	T4	C7-T12	C	E
12	F	4	Falls	60	T10	T10-T12	A	A
13	F	7	Taekwondo	10	T6	T4-T12	B	C
14	M	9	Taekwondo	5	T12	T11-T12	D	E
15	F	3	Falls	10	T8	T6-T12	A	A
16	F	7	Dance	5	T10	T10-T12	C	D

*F* female, *M* male, *Dance* low back exercise during dance, *Taekwondo* lumbar exercise during Taekwondo, *Falls* falling injury from a bed or chair, *C* cervical, *T* thoracic, *L* lumbar.

### Causes of spinal cord injury

Patients in the study group were divided into three subgroups according to the cause of trauma: Dance, Taekwondo, and Falls. A total of eight cases were caused by low back trauma during dance, four cases were attributed to lumbar vertebra trauma during Taekwondo, and four cases were attributed to falling injury from a bed or chair. The latent period from trauma to onset symptoms of spinal cord injuries ranged from 10 minutes to 4 hours (Table 1). We did not observe significant differences in age and gender among the subgroups (*P*>0.05; Table 2). However, the latent period differed significantly among the three subgroups, with Taekwondo having the shortest, followed by falls, and finally dance with the longest period (*P*=0.004; Table 2).

**Table 2:** Assessment of clinical characteristics among the different sub groups

Clinical Characteristics	Dance	Taekwondo	Falls	*P* VALUE
Average age (years)	6.50 (1.69)	4.25 (1.89)	9.50 (2.52)	0.793
M : F	0:8	0:4	1:3	0.212
Latent period (min)	55.00 (83.96)	30.00 (21.60)	35.00 (56.71)	0.007^*^
ISNCSCI				0.715
A	3	3	1	
B	1	1	1	
C	2	0	1	
D	2	0	1	
ISNCSCI after therapy				0.396
A	2	3	1	
B	0	0	0	
C	2	0	1	
D	2	1	0	
E	2	0	2	
MRI				0.039^*^
C and T	1	4	1	
T	4	0	3	
T and L	2	0	0	

*F* female, *M* male, *Dance* low back exercise during dance, *Taekwondo* lumber exercise during Taekwondo, *Falls* falling injury from a bed or chair, *C* cervical, *T* thoracic, *L* lumbar; * : *P*<0.05.

### Clinical manifestation

Among the enrolled patients, none of the patients demonstrated signs of fever, inflammation, tumors, or other pathological conditions that can lead to traumatic injury. All patients presented with back pain or limb pain followed by limb weakness. According to the guidelines from the ISNCSCI, spinal cord injuries were scored on a 5-point scale [12]. In the study group, 10 cases of spinal cord injuries were classified as grade 0 according to the ISNCSCI guidelines, and six cases of paralytic limb muscle strength were classified as grades 1-4.

The ISNCSCI scores indicated pre-school age patients (≤7 years old) had significantly more severe injuries than school age patients (>7 years old) (*P*=0.044; Table 3).

**Table 3:** Clinical characteristics assessment between sub-groups according to different age groups in the study group

Clinical characteristics	Pre-school age	School age	*P* value
Male: Female	0:11	1:4	0.000
ISNCSCI			0.041^*^
A	6	1	
B	2	1	
C	2	1	
D	1	2	
ISNCSCI after therapy			0.002^*^
A	5	1	
B	0	0	
C	2	1	
D	3	0	
E	1	3	
MRI			0.001^*^
C and T	0	2	
T	10	2	
T and L	1	1	

*f* female, *m* male, *PS* pre-school age, *S* school age, *C* cervical, *T* thoracic, *L* lumbar; *:*P*<0.05.

### Laboratory and radiological findings

Routine blood work, C-reactive protein, liver functions, blood lipid, myocardial enzyme, and blood glucose were normal in the study group. The cerebrospinal fluid was also normal in this group.

**Fig. 1:**
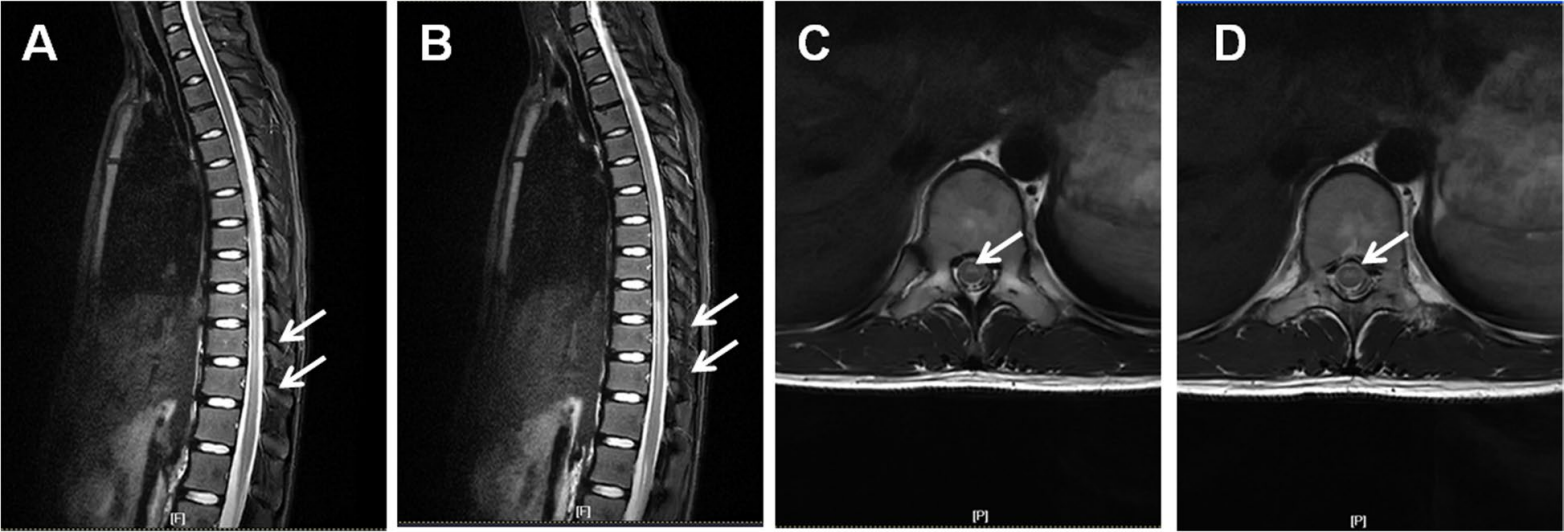
**A**, **B** The sagittal magnetic resonance imaging of the spinal cord in a 9-year-old patient with spinal cord injury (arrow). **C**, **D** Transverse section of the magnetic resonance image of the spinal cord injury (arrow)

No vertebral fractures or dislocations were observed in the study group. However, lesions, similar to the one illustrated in Fig. 1, were in the cervical and thoracic vertebrae, the thoracic vertebrae, and the thoracic and lumbar vertebrae (Table 1). The magnetic resonance images showing spinal cord injury are presented in Fig. 1. Analysis of the imaging findings revealed that the injury level of the spinal cord was significantly different among the pre-school age and school age groups (*P*=0.041; Table 3). The lesions in the pre-school age group were in the thoracic spinal cord, while the lesions in the school age group were in the cervical, thoracic, and lumbar spinal cord.

The spinal cord MRI findings were also significantly different among the three subgroups (*P*=0.041; Table 2). The lesions located in the cervical and thoracic spinal cord were caused from Taekwondo in all four cases. The lesions located in the thoracic spinal cord were caused by low back trauma from dancing in four cases and by falling from a bed or chair in three cases. The lesions located in the lumbar spinal cord were caused by low back trauma from dancing in two cases.

### Treatments and outcomes

All patients were hospitalized for conservative treatments, which included short-term methylprednisolone therapy (10 mg/kg/d for 3 days) and rehabilitation treatment. The ISNCSCI score was not significantly different among the three subgroups pre- or post-therapy (Table 2). However, significant differences in the ISNCSCI grades between the pre-school age (≤7 years old) and school age (>7 years old) patients (*P*=0.002; Table 3) were observed after 1 month of treatment. The ISNCSCI scores were more severe in the pre-school age group (≤7 years old) than in the school age group (>7 years old).

## Discussion

SCIWORA is a relatively rare event in adults but is more common in children, leading to significant disability [13, 14]. SCIWORA is more likely to occur in children under 8 years old. In agreement with the existing data, the mean age of our study group cohort was 6.69±2.51 years. Compared to the school-age patients (>7 years old), our results showed the pre-school age patients (<7 years old) were more vulnerable to SCIWORA, with more severe ISNCSCI.

In China, children begin dance or Taekwondo training at a relatively young age. Accordingly, the causes of trauma in this study were injuries sustained from dance practice, Taekwondo, or falling [3]. Spinal cord injury in the thoracic and thoracolumbar segments is common in SCIWORA cases reported in China [3]. In contrast, in Western countries, SCIWORA cases are mainly due to traffic accidents and the injuries involve the cervical segment [3, 15]. Our results demonstrated that SCIWORA was more common in the thoracic spine. The ribs and abdomen protect the thoracic spine against injury caused by excessive bending and dislocation [16].. However, this protection is ineffective against longitudinal forces caused by extensive forces, which can lead to thoracic spinal cord injuries [17]. Therefore, longitudinal forces produced by excessive extension of the spine can still lead to thoracic spinal cord injury. Further, previous research demonstrated that the age of the patient is associated with the severity of neurological damage [4, 5]. As a child grows, the stability of the spine improves, resulting in a decrease in the incidence of crush injuries to the spinal cord.

In a meta-analysis of 392 SCIWORA cases, 90% of the patients were children and the onset of symptoms ranged from 30 minutes to 48 hours after injury [13, 18]. In our cohort, the latent period for delayed SCIWORA ranged from 10 minutes to 4 hours after injury [19]. Although the neurological deficit can be delayed, it progresses rapidly after commencing [20]. Therefore, high-risk patients should be closely monitored. MRI investigation and careful physical exams are recommended for children with a trauma history once spinal cord injury is suspected [21–23].

Treatment of acute spinal cord injury is mainly based on glucocorticoid impact therapy recommended by the National Acute Spinal Cord Injury Study (NASCIS) [24–27]. Anti-lipid peroxidation and anti-inflammation by glucocorticoids are considered the main mechanisms for their neuroprotective functions. The early application of high-dose methylprednisolone within 8 hours after injury can slow or stop secondary injuries caused by inflammatory reactions resulting from the initial spinal cord injury and improve functional recovery. Children are liable to experience frequent injuries although their spine and ligament repair ability is strong [28]. Severe spinal deformities can develop from SCIWORA. Early intervention may prevent the formation of spinal deformities, and surgical approaches may be the optimal therapeutic choice for deformities resulting from SCIWORA [24, 29]. In agreement with our results, Launay et al. indicated that up to 39% of patients with SCIWORA due to vertebral hyperextension injuries could achieve full recovery [18]

## Conclusion

In conclusion, our results demonstrated that the factors associated with SCIWORA in China included Dance and Taekwondo practices. Spinal cord injury may occur in children after mild trauma, and most injuries are localized to the thoracic spinal cord. SCIWORA should be considered for children with spinal cord injury symptoms following any type of trauma and MRI investigations should be performed. Nevertheless, the relatively small number of cases is the main limitation of this study. This is attributed to the low incidence rate of SCIWORA. Subsequent research is needed to confirm our results.

## Data Availability

The datasets used and/or analyzed during the current study are available from the corresponding author on reasonable request.

## Abbreviations

SCIWORA: Spinal cord injury without radiographic abnormality
ASIA: American Spinal Injury Association
CT: Computed tomography
NASCIS: National Acute Spinal Cord Injury Study
MRI: Magnetic Resonance Imaging
